# Does an Association between the Idiopathic Left-Sided Varicocele and Eye Colour Exist?

**DOI:** 10.1155/2014/524570

**Published:** 2014-04-07

**Authors:** Philip Kumanov, Ralitsa Robeva, Analia Tomova

**Affiliations:** Clinical Center of Endocrinology and Gerontology, Medical University of Sofia, 2 Zdrave Street, 1431 Sofia, Bulgaria

## Abstract

The possible interrelation between male reproductive disorders and iris pigmentation is poorly understood. We have found a link between eye colour and the existence of adolescent varicocele. Therefore, we aimed to extend our investigation on the relationship between the eye colour and varicocele in adult men. 231 andrology outpatients from Caucasian origin were included in the study. The presence of varicocele, sperm disturbances, and the iris pigment of the patients were investigated. Left-sided varicocele was found in 93 adults. In the group of light-eyed men the prevalence of varicocele was significantly lower than among the dark-eyed men (15% versus 59.5%, *P* < 0.001). No associations were found between the eye colour and disturbances in semen parameters in males with varicocele (*P* = 0.419) and in those without varicocele (*P* = 0.586). The present results in adult men suggest that the prevalence of varicocele could be associated with the iris pigment. A possible genetic linkage between the eye colour and the susceptibility to some disorders like varicocele could not be excluded. However, the iris pigmentation seems not to have a direct relationship with the sperm disturbances.

## 1. Introduction 

Idiopathic varicocele is an abnormal dilation of the veins of the pampiniform plexus of the spermatic cord, usually left-sided, and represents the most common cause for male infertility that could be identified and successfully treated [1]. Our previous data showed that serum inhibin B levels were significantly lower in the varicocele group, compared with the controls, suggesting disturbances of Sertoli cells caused by varicocele [2]. Leydig cell function can be also impaired. The underlying mechanisms of the negative effects of varicocele on testicular function are still unknown, but hypotheses include venous stasis, increased testicular temperature, oxidative stress, and resulting toxic environment [3]. Considering the clinical significance of varicocele, it is important to identify the risk factors for the disease. On the other hand there are some data on the possible association of the eye colour and some diseases in both animals and humans [4–6]. According to our previous study idiopathic left-sided varicocele was found significantly more often among dark-eyed adolescent boys (10–19 years) compared to light-eyed coevals [7]. Therefore, we aimed to investigate whether the pigmentation of the iris could be related to this venous abnormality development also in adults.

## 2. Methods

In this prospective study 231 Caucasian males (mean age 32.91 ± 9.31 years) from all strata of the society, visiting our outpatient andrology clinic with different complaints including erectile dysfunction, infertility due to various causes, metabolic syndrome, diabetes type 2, and hypertension, were examined at random. Physical examination for varicocele was done by one investigator (PhK) by palpating the spermatic cord in a standing position before and during the Valsalva maneuver. The varicocele presence was proved by ultrasonography. The iris pigment of the patients was also described. Eye colors were divided into two categories: dark-eyed (black-brown and brown) and light-eyed (green-brown, gray-green, and blue). In the whole investigated group 56.7% (*n* = 131) were dark-eyed and 43.3% (*n* = 100) were light-eyed as expected in our population [8]. Semen samples were obtained from 52 patients with and from 83 men without varicocele and the semen analyses were done according to WHO laboratory manual for the examination and processing of human semen, fifth edition, 2010 [9]. The present study was conducted according to the Helsinki declaration and all participants gave their informed consent.

The statistical analyses were performed with SPSS for Windows 11.0 (SPSS Inc., Chicago, IL). After the Kolmogorov-Smirnov test for normality of distribution Student's *t*-test was done. Frequency analyses, descriptive statistics, *χ* ^2^ test, Fisher's exact test, and logistic regression were used where appropriate. A *P* value of less than 0.05 was considered statistically significant.

## 3. Results

**Figure 1:**
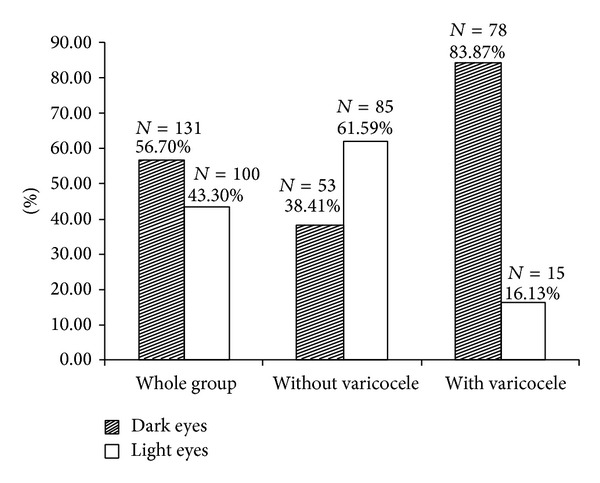
Percent of the men with dark and light eyes in the whole group and in patients with and without varicocele.

93 (40.26%) of 231 investigated men were with left-sided varicocele. 83.87% (*N* = 78) from the patients with varicocele were with dark eyes and 16.13% (*N* = 15) with light-colored eyes (Figure 1). Considering the eye color, the prevalence of varicocele in the group of the light-eyed men was significantly lower than in those with dark-pigmented eyes (15% versus 59.5%, *P* < 0.001). The patients with varicocele were younger than the others (30.63 ± 8.12 versus 34.44 ± 9.77 years, *P* = 0.002), but even after adjustment for age the risk for varicocele development was strongly increased in dark-eyed men compared to the light-eyed men, odds ratio 8.234, 95% CI (4.251–15.951), *P* < 0.001. No associations were found between the eye color and disturbances in semen parameters (sperm volume, sperm count and density, and percentage of the progressive motile sperms) in males with varicocele and in the whole investigated group (Table 1).

## 4. Discussion

The present results in adult men support our previous finding in adolescents that light iris pigment could be associated with a lower prevalence of varicocele [7]. The stronger association found in the present study as well as the different prevalence of varicocele in both studies could be explained with the pronounced methodological differences. Only men with overt clinical complaints (actively seeking andrological help) were included in the present study, while varicocele was accidentally found during clinical examinations of adolescent boys in a population-based study focused on the normal growth and pubertal development.

Several interrelations between the eye colors and some diseases were already reported in animals and humans [4–6]. Significantly more diabetic patients in southern Germany had blue eyes than nondiabetic control subjects, while the macular edema was more prevalent in blue- or grey-eyed diabetic persons in comparison to those with intermediate or brown eyes [4, 5]. Eleven percent from Italian women with rectovaginal endometriosis were with green or blue eyes, while only 3% of healthy controls were with light eyes [10].

The association between the iris color and varicocele could result from a genetic linkage. According to the principles of phenomics, phenotypic information could be used in conjunction with genetics and environmental condition to reveal the pathogenesis of different disorders [10]. Human iris pigment was strongly related to mutations in the long arm of chromosome 15 [11–13]. Large investigations had shown that several single nucleotide polymorphisms in the oculocutaneous albinism type II gene (OCAII) and the neighboring HERC2 gene were strongly related to the human iris pigment [11–13]. Different genetic variants of HERC2 or mutations within the 11.7 kb of sequence between the OCA2 and HERC2 genes might regulate the expression of OCAII gene [12]. Beyond the iris color, mutations in OCAII gene encoding the human pigment protein were associated with a wide range of hypopigmented states, for example, type II oculocutaneous albinism, hypopigmentation in Prader-Willi, and Angelman syndrome [13]. The complex phenotype of the patients with Prader-Willi syndrome is due to the loss of expression of several genes on chromosome 15 and includes different clinical signs beyond hypopigmentation as severe obesity, disturbed sense of satiety, hypogonadotrophic hypogonadism, and so forth [14]. The development of varicocele has also a strong genetic basis, and increased prevalence is found in the first-degree relatives of patients with known varicocele [15].

According to Eiberg et al. [16] blue eye color in humans was caused by a founder mutation with a subsequent positive selection for this phenotype leading to a very rapid increase in the prevalence of people with light iris color in Europe [16]. According to Frost [17] the increased attractiveness of light-eyed women and presumed higher estrogenicity were suggested as a logical explanation for better mating chances and reproductive potential of the ancient women [17, 18]. However, male attractiveness was not related to the eye color. On the contrary, blue-eyed men were rated as less dominant than brown-eyed men, a finding that could influence rather negatively the distribution of the light eyes genotypes [19]. Since sexual selection could not fully explain the eye-colour diversity, genetic linkage between light iris pigment and other physiological features might be expected. Increased frequency of light-eyed men could be due to some unknown factors related to better reproductive chances in the ancient environment. The lower frequency of varicocele in light-eyed European ancestors might be an important feature related to better sperm indices and consequently to more children. Rapid pubertal development is an important negative factor for the development of varicocele, whereas increased weight shows a protective role [7]. Strong physical activity is related to significant sperm impairment in men with varicocele [20]. In our study, we did not find any differences in the sperm indices between light-eyed and dark-eyed men. However, in the ancient times, when the strong physical activity was mandatory for survival and the prevalence of obesity was extremely low, light-eyed ancestors with lower varicocele prevalence might have sustained better sperm indices and higher reproductive potential than dark-eyed ones. Linkage equilibrium between genes responsible for eye color and genes related to varicocele could explain the process of light-eye positive selection. Future research could reveal whether the association of dark eyes and left-sided varicocele is valid in other ethnic groups or it is specific for our population.

Another interesting question is whether the eye color is related only to the presence of varicocele or it could be associated with other diseases characterized by a general venous weakness, for example, in the lower extremities.

## Figures and Tables

**Table 1:** Associations between sperm disturbances and eye color in men with or without varicocele.

	Men with varicocele		Men without varicocele	
	Normal sperm	Sperm disturbances	Normal sperm	Sperm disturbances
Dark-eyed men	25.6%	74.4%	16.7%	83.3%
Light-eyed men	44.4%	55.6%	23.4%	76.6%
*P*	**0.419**		**0.586**	
